# Effects of a Hypocaloric Diet and Physical Training on Ventilatory Efficiency in Women with Metabolic Syndrome: A Prospective Interventional Study

**DOI:** 10.3390/ijerph22101520

**Published:** 2025-10-03

**Authors:** Caroline Simões Teixeira, Débora Dias Ferraretto Moura Rocco, Raphael de Souza Pinto, Alexandre Galvão da Silva, Alessandra Medeiros

**Affiliations:** 1 Physiology Laboratory of Health, Santa Cecília University, Santos 11045-907, Brazil; 2 Biomedical Faculty, São Caetano University, São Caetano 09521-310, Brazil; 3 Department of Bioscience, Universidade Federal de São Paulo (Unifesp), Santos 11015-020, Brazil; a.medeiros@unifesp.br

**Keywords:** metabolic syndrome X, respiratory function test, exercise therapy, diet

## Abstract

Metabolic syndrome (MetS) is a multifactorial clinical condition characterized by the co-occurrence of abdominal obesity, impaired glucose metabolism, high blood pressure, and dyslipidemia. Non-pharmacological strategies, such as hypocaloric diets (HD) and structured physical training (PT), have shown promise in improving metabolic and functional outcomes in this population. The aim of this prospective interventional study was to evaluate the effects of a 16-week program combining HD with PT on ventilatory efficiency and cardiometabolic risk markers in women with MetS. Forty-one sedentary women (aged 45–55 years) with clinically diagnosed MetS underwent anthropometric, metabolic, nutritional, and cardiopulmonary assessments before and after the intervention. Participants engaged in 60 min exercise sessions three times per week and followed a personalized hypocaloric diet targeting 5–10% weight loss. Post-intervention analyses revealed significant reductions (*p* ≤ 0.05) in body weight (from 86.6 kg ± 3.3 kg to 78.2 kg ± 3.3 kg), body fat percentage (40.1% ± 0.6% to 33.4% ± 1.6%), and waist circumference (105.1 cm ± 1.2 cm to 95.7 cm ± 1.9 cm). Improvements were also observed in fasting glucose (from 117.1 mg/dL to 95.1 mg/dL) and triglycerides (158.8 mg/dL ± 9.1 mg/dL to 111.8 mg/dL ± 9.1 mg/dL), and in lean mass percentage (59.9% ± 6.5% to 66.6% ± 1.7%). Cardiopulmonary variables showed enhanced ventilatory function, with increased VO_2_peak (1.59 L/min ± 0.1 L/min to 1.74 ± 0.1 L/min), improved oxygen uptake efficiency slope (OUES), and a steeper VO_2_/workload relationship. Resting heart rate and blood pressure declined significantly (69.9 bpm ± 2.0 bpm to 64.9 ± 1.8 bpm; 145.4 mmHg ± 3.9/80.2 ± 3.0 mmHg to 140.1 mmHg ± 2.7/75.2 ± 1.6 mmHg). In conclusion, the 16-week intervention combining HD with PT proved effective for reducing cardiometabolic risk factors and enhancing ventilatory efficiency, suggesting improved integration of oxygen uptake, transport, and utilization in the women with MetS assessed.

## 1. Introduction

We are witnessing substantial epidemiological changes in health due to shifts in lifestyles adopted by societies in recent decades, directly reflected by a transformation in causes of illness and death [1]. According to the World Health Organization (WHO), chronic non-communicable diseases, including cardiovascular conditions, are responsible for approximately two-thirds of all global deaths [2]. In this context, metabolic syndrome (MetS) emerges as a complex cluster of interrelated clinical conditions that substantially increases cardiovascular risk. MetS can be defined according to different diagnostic criteria, but the core components of the syndrome include abdominal obesity, impaired glucose metabolism, arterial hypertension, and dyslipidemia [3]. These factors are often associated with low physical activity and unhealthy lifestyle habits.

Evidence indicates that insulin resistance and inflammatory processes resulting from visceral obesity play a central role in the pathophysiology of MetS [4]. Excessive accumulation of adipose tissue promotes metabolic dysfunctions that result in lipotoxicity, peripheral insulin resistance, and low-grade chronic inflammation involving macrophages and pro-inflammatory cytokines [5]. Additionally, dysfunction of the central nervous system in nutrient sensing and appetite regulation further contributes to obesity and impaired glycemic control, establishing a vicious cycle between insulin resistance and hyperinsulinemia.

Despite the growing body of knowledge about the deleterious effects of MetS on cardiovascular and respiratory systems [6], the prevalence of the syndrome continues to increase amid rising rates of obesity and type 2 diabetes mellitus [7].

There is evidence of an inverse relationship between physical fitness and MetS prevalence, suggesting that high levels of cardiorespiratory fitness represent an important protective factor [8]. Maximal oxygen uptake (VO_2_max) has consistently been identified as an independent prognostic indicator of cardiovascular and all-cause mortality [8].

Church (2011) [8] suggests that increments in cardiorespiratory fitness, often quantified in 1 MET increases, are associated with a 10–15% relative risk reduction in cardiovascular events. However, these associations are predominantly correlational, while causality cannot be inferred without considering confounding variables, such as baseline fitness level, comorbidities, and intervention type [8].

Teixeira et al. (2021) [9] demonstrated that individuals with MetS exhibit impaired cardiorespiratory efficiency during maximal physical exertion, as assessed by parameters such as VO_2_max and anaerobic threshold (VO_2_AT). These findings support the hypothesis that factors associated with MetS negatively impact cardiorespiratory performance, directly affecting functional capacity and exercise response [9].

Consequently, non-pharmacological therapeutic strategies have been widely recommended, with emphasis on the combination of a hypocaloric diet and regular physical activity. Such interventions have shown synergistic effects in reducing body mass and improving metabolic, cardiovascular, and autonomic parameters [10].

Ventilatory and metabolic parameters derived from cardiopulmonary exercise testing (CPET) offer insights into the interplay between respiratory, cardiovascular, and muscular systems under physiological stress. Of these parameters, the oxygen uptake efficiency slope (OUES), a submaximal index obtained from the logarithmic relationship between oxygen uptake (VO_2_) and ventilatory equivalence (VE), or minute ventilation, has emerged as a reliable marker of ventilatory efficiency and cardiorespiratory reserve [11]. While originally applied in chronic cardiopulmonary conditions, recent evidence suggests that individuals with MetS also exhibit reduced OUES, likely driven by the burden of central adiposity, low-grade systemic inflammation, and altered respiratory mechanics [12]. Ventilatory equivalence, defined as the total volume of air exhaled per minute, normally increases proportionally to exercise intensity. In MetS, however, increased thoracoabdominal impedance and ventilatory inefficiency may lead to exaggerated ventilatory responses even at submaximal workloads, compromising gas exchange and increasing perceived exertion [13]. Structured aerobic training and hypocaloric diet (HD) interventions have been shown to attenuate these impairments by reducing body mass, improving ventilatory patterning, and enhancing respiratory muscle efficiency [14]. Importantly, these interventions also promote systemic metabolic adaptations that may contribute to an overall reduction in cardiometabolic risk in MetS, beyond improving ventilatory parameters alone.

Recent randomized controlled trials have confirmed the effectiveness of hypocaloric diets in reducing visceral adiposity, improving insulin sensitivity, and lowering both triglyceride levels and blood pressure in affected individuals [15]. Moreover, dietary interventions involving calorie restriction have been shown to significantly enhance metabolic outcomes and reduce cardiometabolic risk. These effects are largely attributed to reductions in systemic inflammation, improvements in adipokine profiles, and enhanced mitochondrial efficiency. However, Valsdottir et al. (2020) demonstrated that an intervention with diet alone (as opposed to physical training) promoted a reduction in fat mass but no impact on cardiorespiratory fitness [16].

The growing prevalence of MetS and its association with impaired cardiorespiratory function underscore the need for effective non-pharmacological strategies. While physical training and hypocaloric diets have demonstrated benefits in improving metabolic and anthropometric parameters, their specific influence on ventilatory efficiency, an important predictor of functional capacity and clinical prognosis, remains underexplored [9].

This study seeks to address this gap by evaluating the effects of a combined intervention on ventilatory, metabolic, and anthropometric responses in women with MetS. The focus on the female population is justified by both the relative scarcity of studies involving females compared to males and the known pathophysiological differences between sexes, especially in the context of chronic inflammation commonly observed in individuals with overweight and obesity. The study supports the hypothesis that lifestyle changes, in the form of a low-calorie diet and physical training, are effective in benefiting the cardiopulmonary system, as well as favoring metabolic variables and weight reduction.

Thus, the objective of this study was to investigate the effects of a physical training program, combined with a hypocaloric diet, on ventilatory efficiency during maximal progressive exercise, as well as on modulating anthropometric and metabolic variables among women with MetS.

## 2. Materials and Methods

### 2.1. Participants

Forty-one female patients aged 45–55 years with a clinical diagnosis of metabolic syndrome (MetS) were evaluated. These individuals attended the PROCardio Rehabilitation Clinic and the Laboratory of Exercise Physiology and Health (LAFES) at Universidade Santa Cecília (UNISANTA) for medical consultations and physical assessments. Patients meeting the inclusion and exclusion criteria were invited to participate and provided written informed consent. All data collection procedures followed the CONSORT guidelines for clinical trials [17].

Sample size calculation was performed using G*Power software (version 3.1.9.7), based on an effect size of 0.8, alpha of 0.05, and power of 0.80, indicating a minimum of 34 participants. To reduce potential bias, all measurements were made by the same evaluator, and standardized protocols were followed for each test.

Participants underwent medical evaluation and assessments of blood pressure, body weight, body mass index (BMI), waist circumference, laboratory tests (serum levels of total cholesterol, triglycerides, HDL-cholesterol, and fasting glucose), and cardiopulmonary exercise testing. Resting blood pressure was measured in a seated position, after 5 min of rest, using a validated digital sphygmomanometer (Omron HEM-7120, Kyoto, Japan), in accordance with the guidelines of the Brazilian Society of Cardiology [18].

### 2.2. Study Design and Ethical Approval

This study was approved by the Research Ethics Committee of the Federal University of São Paulo (UNIFESP), Santos—SP (CAAE: 64535922.8.0000.5505), under approval number 5.821.626 and registered on the Brazilian Registry of Clinical Trials (ReBEC) platform under the registration number RBR-96hpf7j.

To be eligible for the study, participants had to be sedentary and present at least three of the following criteria as defined by the National Cholesterol Education Program (2002) [19]: abdominal circumference ≥ 102 cm for men and ≥88 cm for women; triglycerides ≥ 150 mg/dL; HDL-cholesterol < 40 mg/dL for men and <50 mg/dL for women; systolic blood pressure ≥ 130 mmHg and/or diastolic blood pressure ≥ 85 mmHg; and fasting glucose ≥ 100 mg/dL. Exclusion criteria included smoking, alcohol misuse, a diagnosis of neoplasms in the last five years, or attending < 75% of the training sessions and/or nutritional consultations.

Following the initial assessments, all participants received individualized nutritional guidance and follow-up every two weeks with a registered dietitian. The intervention involved a hypocaloric diet aiming to achieve a 5–10% reduction in initial body weight. All assessments (including medical consultations) were performed both before and after the 16-week intervention period.

### 2.3. Body Composition Assessment

Body composition was evaluated using the bioimpedance method, which relies on the electrical properties of tissues to assess fat and lean mass.

Measurements were performed using a tetrapolar bioimpedance scale (InBody 770, Seoul, Republic of Korea), following standardized pre-test conditions: 12 h fasting, no exercise for 24 h, and bladder voided before testing. Additional measurements included body weight, height, BMI calculation, and waist circumference.

### 2.4. Laboratory Tests

Blood tests were conducted at the PROCardio Clinic and analyzed by the Delboni Auriemo Clinical Analysis Laboratory. During the morning period, after a 12 h fast, 4.5 mL of venous blood was collected from the antecubital vein of patients. Fasting glucose was measured using an enzymatic automated method (Roche). Lipid profile assessment included total cholesterol, triglycerides, and HDL-cholesterol using a colorimetric enzymatic method, and LDL-cholesterol was calculated according to the IV Brazilian Guidelines on Dyslipidemia of the Brazilian Society of Cardiology (2021) [20].

### 2.5. Cardiopulmonary Exercise Test

The cardiopulmonary exercise test (CPET) was performed using the SensorMedics Vmax Analyzer Assembly, Encore 29S. Data were collected breath-by-breath and averaged over 30 s intervals. The following parameters were analyzed: oxygen consumption (VO_2_, L/min or mL/kg/min, STPD); carbon dioxide production (VCO_2_, mL/min, STPD); pulmonary ventilation (VE, L/min, BTPS); tidal volume (Vt, L/min, BTPS); respiratory rate (RR, breaths/min); estimated functional dead space (VD/VT); respiratory exchange ratio (RER); ventilatory equivalents for oxygen (VE/VO_2_) and carbon dioxide (VE/VCO_2_); and end-tidal partial pressures of oxygen and carbon dioxide (PetO_2_ and PetCO_2_, mmHg). Prior to each evaluation, the metabolic analyzer was calibrated with certified gas mixtures of CO_2_ and O_2_ balanced with nitrogen, and the flow meter was calibrated using a 3 L syringe.

The test was performed on an electromagnetically braked cycle ergometer (CardioControl, Lode BV, Groningen, The Netherlands) using a ramp protocol with continuous load increments (5–20 W/min) and constant pedaling cadence between 60 and 70 rpm until volitional exhaustion. Heart rate behavior was monitored via a standard 12-lead ECG (Marquette Medical Systems, Inc. CardioSoft, Milwaukee, WI, USA), and blood pressure was assessed by auscultation. The test was considered maximal when the respiratory exchange ratio reached ≥1.10, along with patient-reported exhaustion [21]. Physical capacity was determined by peak oxygen consumption at the end of the test. ECG tracings and blood pressure readings were recorded at rest, during exercise, and during recovery. Participants were also asked periodically about symptoms such as fatigue, leg heaviness, and dizziness.

### 2.6. Oxygen Uptake Efficiency Slope

Oxygen uptake efficiency slope (OUES) was calculated during CPET using the linear relationship between VO_2_ (mL/min) and VE (L/min) as described by Baba et al. (1996) [22], using the equation *y* = *ax* + *b*, where *y* = VO_2_; *a* = rate of increase in VO_2_ in response to VE; *b* = intercept (VO_2_ at rest); and *x* = VE transformed using a base-10 logarithm to linearize its behavior during maximal exertion. The constant *a* was used to compute the OUES. OUES was assessed at 75%, 90%, and 100% of maximum effort time [23], and expressed both as absolute values (mL/min) and values relative to body weight (mL/kg/min) [24]. Predicted values were calculated using Hollenberg’s formula (2000) [23] for men: OUES (L/min) = [1320 − (26.7 × age) + (1394 × body surface area)]/1000; and for women: OUES (L/min) = [1175 − (15.8 × age) + (841 × body surface area)]/1000; Oxygen Uptake-to-Workload Ratio (VO_2_/W).

The VO_2_/W slope was assessed during the CPET using the linear regression between VO_2_ and workload (Watts) applying the equation *y* = *ax* + *b*, where *y* = VO_2_; *a* = increase in VO_2_ per unit of workload; *b* = intercept (VO_2_ at rest); *x* = workload in Watts [25]. The slope was analyzed both from the first minute of exercise to the respiratory compensation point (RCP) and from the first minute to peak exercise. RCP was determined by at least one of the following criteria: (1) a peak in PetCO_2_ followed by a sharp decline; (2) loss of linearity in the VE vs. VCO_2_ relationship as evidenced by the VE/VCO_2_ slope [26]. VO_2_ was analyzed in both absolute terms (mL/min) and relative to body weight (mL/kg/min).

### 2.7. Ventilatory Anaerobic Threshold

Ventilatory anaerobic threshold (VAT) was identified using the V-slope method [15], which detects the breakpoint in the linear relationship between VCO_2_ and VO_2_. Additional conventional criteria were employed, including increases in VE/VO_2_ and PetO_2_, along with stable VE/VCO_2_ and PetCO_2_ values [27].

### 2.8. Physical Training Program

The exercise program lasted 16 weeks, with sessions conducted three times per week. Each session had a duration of 60 min and comprised 5 min of warm-up, 45 min of aerobic exercise (treadmill or cycle ergometer, depending on availability and participant preference), 10 min of resistance exercises targeting two muscle groups (one upper and one lower limb group per session, alternating weekly to ensure comprehensive training), and 5 min of relaxation [28].

Exercise intensity during the 16-week protocol was divided into three progressive stages: (1) the first 10 sessions were performed at the anaerobic threshold (AT); (2) the subsequent 16 sessions (sessions 11–26) were conducted at intensities between AT and the respiratory compensation point (RCP); and (3) the final 22 sessions (sessions 27–48) were performed near RCP. Intensity was monitored using heart rate obtained during the CPET. When training was conducted on a cycle ergometer, heart rate was adjusted to 10% below the value corresponding to each threshold.

### 2.9. Hypocaloric Diet Protocol

Participants followed a low-glycemic-index hypocaloric diet (500 kcal deficit) throughout the intervention, targeting a weight loss of 5–10% total body weight. At baseline, a dietary history was taken using specialized nutrition software to assess habitual intake. The diet was distributed across five meals per day, with macronutrient composition comprising 55–75% carbohydrates, 10–15% protein, and 15–30% fat [20]. Simple sugars and foods high in added sugar were excluded. Adherence was monitored through biweekly consultations, body weight assessments, and food diary reviews.

### 2.10. Statistical Analysis

Data are expressed as mean ± standard deviation. The Kolmogorov–Smirnov test was applied to the study variables to assess normality. Variables not adhering to a normal distribution were log-transformed prior to analysis.

Anthropometric, metabolic, and ventilatory variables were analyzed using one-way and two-way repeated measures analysis of variance (ANOVA). When a significant F-ratio was observed, post hoc comparisons were conducted using Scheffé’s test. The level of statistical significance was set at *p* < 0.05.

## 3. Results

Of the 64 women initially recruited, only 41 met all the inclusion and exclusion criteria and completed the intervention. The mean age of the participants was 51.4 ± 6 years. The anthropometric and metabolic characteristics of the participants, before and after the 16-week intervention, are presented in Table 1. Following the intervention, all anthropometric characteristics showed a significant reduction (including weight, BMI, waist circumference, and fat mass), whereas lean mass significantly increased after the intervention period. Regarding lipid parameters, a significant reduction was observed in fasting glucose and triglyceride concentrations only.

The results presented in Table 2 show a significant decrease in systolic and diastolic blood pressures, as well as in resting heart rate, following the intervention of physical training plus hypocaloric diet.

**Figure 1:**
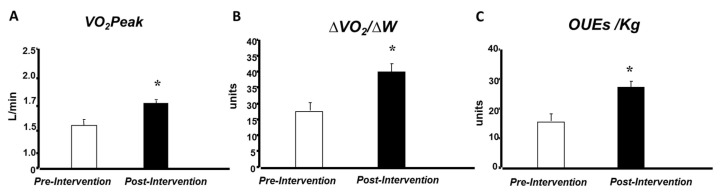
Peak oxygen consumption (**A**), workload slope (**B**), and oxygen uptake efficiency slope (**C**), before and after intervention period. * *p* ≤ 0.05 for pre- vs. post-intervention comparison.

Figure 1 illustrates the pre- and post-intervention values for VO_2_peak (1.59 ± 0.1 to 1.74 ± 0.1 L/min), workload slope (∆VO_2_/∆W), and oxygen uptake efficiency slope (OUES), corrected for body weight, are illustrated in Figure 1. Notably, values of all variables increased following the intervention period.

## 4. Discussion

The results of this study revealed that a 16-week combined intervention of hypocaloric diet and physical training led to significant improvements in ventilatory efficiency, cardiorespiratory capacity, and metabolic parameters in women with metabolic syndrome (MetS). Anthropometric markers such as body weight, BMI, body fat percentage, and waist circumference were significantly reduced (*p* ≤ 0.05), indicating the potential responsiveness to the intervention of the intervention in improving metabolic health. These outcomes are consistent with previous evidence linking abdominal adiposity to systemic inflammation and metabolic dysregulation [29].

Although body composition was not the primary outcome of this study, the reduction in waist circumference observed is relevant due to its strong association with cardiometabolic risk. In a recent population-based study in India, the prevalence of MetS among individuals with abdominal circumference above reference values was 73.8%, compared to 20.4% in participants whose values were within reference limits [30]. This reinforces the clinical importance of abdominal adiposity control as part of lifestyle interventions.

In addition to the anthropometric changes in the present investigation, improvements in glycemic and lipid profiles were observed, including a 15% reduction in fasting glucose, besides favorable changes in HDL-c and triglycerides. While these findings are well established in the literature, they serve to corroborate the physiological pathways through which physical training and dietary control indirectly enhance ventilatory and cardiovascular efficiency. Aerobic exercise increases insulin sensitivity and endothelial function, contributing to improved blood pressure and metabolic control [31].

Importantly, the present study showed significant increases in VO_2_peak and submaximal ventilatory parameters, aligning with the main objective of evaluating ventilatory efficiency in women with MetS. VO_2_peak reflects the integrative capacity of the cardiovascular, respiratory, and muscular systems, where increased VO_2_peak is associated with improved survival and reduced cardiovascular events [32].

VO_2_peak is influenced by volitional effort, and its use may face limitations in clinical populations. This study highlights the role of submaximal indicators such as oxygen uptake efficiency slope (OUES) and ∆VO_2_/∆W ratio. OUES reflects the relationship between VO_2_ and minute ventilation (VE) and is less effort-dependent, providing a more reliable index of cardiorespiratory function [22].

Improvements in OUES post-intervention indicate enhanced oxygen uptake and reduced ventilatory effort per unit of oxygen consumed, revealing improved physiological efficiency of the cardiorespiratory system. In a study with obese women, Onofre et al. emphasized the utility of OUES for evaluating functional capacity [33].

In this study, ∆VO_2_/∆W also increased after the intervention, suggesting improved metabolic efficiency and oxygen extraction during progressive exercise. While Hansen et al. (1987) [25] first demonstrated this parameter’s clinical relevance in cardiovascular and mitochondrial disorders [34], more recent studies support its use for assessing functional improvement in chronic metabolic conditions [35].

These findings indicate that, as oxygen transport and utilization become more efficient, individuals with MetS may perform physical tasks with reduced ventilatory demand, delaying the onset of fatigue and enhancing exercise tolerance. This physiological adaptation is particularly valuable for populations with impaired metabolic flexibility and exercise intolerance.

OUES and ∆VO_2_/∆W have attracted increasing interest in clinical practice due to their lower susceptibility to methodological errors (such as those associated with VO_2_peak), while still providing valuable measures of cardiorespiratory fitness [36].

## 5. Conclusions

The present study showed that a 16-week intervention combining a hypocaloric diet with physical training significantly improved ventilatory efficiency, metabolic markers, and anthropometric parameters in women with metabolic syndrome. These findings reinforce the physiological benefits of structured lifestyle modifications in reducing cardiovascular risk.

However, the study has some limitations, including the absence of a control group, short follow-up duration, and a homogeneous sample limited to middle-aged women, potentially restricting the generalizability of results. Despite these limitations, the results suggest that lifestyle interventions should be considered as a first-line strategy in the management and prevention of metabolic syndrome, particularly due to their feasibility, safety, and positive impact on oxygen utilization, cardiopulmonary performance, and metabolic health. Future studies should explore long-term outcomes and the applicability of similar models in different populations.

## Figures and Tables

**Table 1:** Anthropometric and metabolic characteristics of participants, before and after the intervention period.

Variable	Pre-Intervention	Post-Intervention
Weight (kg)	86.6 ± 3.3	78.2 ± 3.3 *
BMI (kg/m^2^)	31.9 ± 0.6	28.9 ± 0.6 *
Fat (%)	40.1 ± 0.6	33.4 ± 1.6 *
Lean mass (%)	59.9 ± 6.5	66.6 ± 1.7 *
WC (cm)	105.1 ± 1.2	95.7 ± 1.9 *
FG (mmol/L)	6.5 ± 0.2	5.3 ± 0.2 *
TC (mmol/L)	5.4 ± 0.3	4.7 ± 0.3
LDL-c (mmol/L)	3.4 ± 0.3	2.9 ± 0.2
HDL-c (mmol/L)	1.0 ± 0.1	1.2 ± 0.1
TG (mg/dL)	1.8 ± 0.1	1.3 ± 0.91 *

Values expressed as mean ± standard deviation. BMI—body mass index; WC—waist circumference; FG—fasting glucose; TC—total cholesterol; LDL-c—low-density lipoprotein cholesterol; HDL-c—high-density lipoprotein cholesterol; TG—Triglycerides. * *p* ≤ 0.05 for pre- vs. post-intervention comparison.

**Table 2:** Hemodynamic parameters at rest before and after intervention period.

Variables	Pre-Intervention	Post-Intervention
SBP (mmHg)	145.4 ± 3.9	140.1 ± 2.7 *
DBP (mmHg)	80.2 ± 3.0	75.2 ± 1.6 *
HR (bpm)	69.9 ± 2.0	64.9 ± 1.8 *

Values expressed as mean ± standard deviation. SBP—Systolic Blood Pressure; DBP—Diastolic Blood Pressure; HR—Heart Rate. * *p* ≤ 0.05 for pre- vs. post-intervention comparison.

## Data Availability

The data generated during the current study are available from the corresponding author on reasonable request.

## Abbreviations

The following abbreviations are used in this manuscript:

MetS	Metabolic syndrome
HD	Hypocaloric diet
PT	Physical training
CPET	Cardiopulmonary exercise test
OUES	Oxygen uptake efficiency slope
VO_2_/W	Oxygen uptake-to-workload ratio
